# Validation of CASPRI, GO-FAR, PIHCA scores in predicting favorable neurological outcomes after in-hospital cardiac arrest; A five-year three center retrospective study in IRAN

**DOI:** 10.1186/s12872-024-04229-8

**Published:** 2024-10-29

**Authors:** Fatemeh safari Alamuti, Seyedehzahra Hosseinigolafshani, Mehdi Ranjbaran, Leili Yekefallah

**Affiliations:** 1 Qazvin University of Medical Sciences, Qazvin, Iran; 2 Social Determinants of Health Research Center, , Research Institute for Prevention of Non-Communicable Diseases, Qazvin University of Medical Sciences, Qazvin, Iran; 3 Non-Communicable Diseases Research Center, Research Institute for Prevention of Non-Communicable Diseases, Qazvin University of Medical Sciences, Qazvin, Iran

**Keywords:** Validation, Cardiac arrest, Cardiopulmonary resuscitation, Neurological outcomes, CASPERI

## Abstract

**Background:**

Predicting neurological outcomes following in-hospital cardiac arrest is crucial for guiding subsequent clinical treatments. This study seeks to validate the effectiveness of the CASPRI, GO-FAR, and PIHCA tools in predicting favorable neurological outcomes after in-hospital cardiac arrest.

**Method:**

This retrospective study utilized a Utstein-style structured form to review the medical records of patients who experienced in-hospital cardiac arrest between March 2018 and March 2023. Predictors were examined using multivariable logistic regression, and the validity of the tools was assessed using ROC curves. Statistical analysis was conducted using SPSS version 25 software.

**Results:**

Out of the 1100 patients included in the study, 42 individuals (3.8%) achieved a favorable neurological outcome. multivariable regression analysis revealed that age, respiratory failure, resuscitation shift, duration of renal failure, and CPC score 24 h before cardiac arrest were significantly associated with favorable neurological outcomes. The predictive abilities of the CASPRI, GO-FAR, and PIHCA scores were calculated as 0.99 (95% CI, 0.98–1.00), 0.98 (95% CI, 0.97–0.99), and 0.96 (95% CI, 0.94–0.99) respectively. A statistically significant difference was observed in the predictive abilities of the CASPRI and PIHCA scores (*P* = 0.001), while the difference between CASPRI and GO-FAR did not reach significance (*P* = 0.057). Additionally, there was no significant difference between the predictive abilities of GO-FAR and PIHCA scores (*P* = 0.159).

**Conclusion:**

The study concludes that CASPRI and GO-FAR scores show strong potential as objective measures for predicting favorable neurological outcomes post-cardiac arrest. Integrating these scores into clinical decision-making may enhance treatment and care strategies, in the Iranian healthcare context.

**Supplementary Information:**

The online version contains supplementary material available at 10.1186/s12872-024-04229-8.

## Background

Cardiac arrest occurs when the heart suddenly ceases to function, resulting in a cessation of breathing and consciousness [1]. It is a major global health concern, contributing to an estimated 17.8 million deaths annually worldwide [2]. In the United States alone, the annual incidence of cardiac arrest in adults is approximately 292,000 cases [3]. According to the latest report from the Iranian Statistics Center, the death toll attributed to cardiac arrest in the first six months of 2023 is approximately 208,000 cases (https://www.amar.org.ir/). Cardiac arrest can manifest in various settings, including public spaces, households, and healthcare facilities. When it occurs within a hospital setting, it is referred to as in-hospital cardiac arrest (IHCA).

IHCA represents a potentially life-threatening event, occurring at a rate of 3 to 6 cases per 1,000 hospital admissions in the United States. Despite its significant impact on healthcare, research interest in IHCA lags behind that of out-of-hospital cardiac arrest (OHCA) [4]. Hospitalized patients, particularly those with severe clinical conditions, are under continuous monitoring, enabling the prediction and prevention of most IHCA cases compared to OHCA [5]. As a result, spontaneous return of blood circulation is anticipated in approximately 70–75% of IHCA cases [6].

Survival rates following IHCA exhibit global variation. Approximately 24% of patients who achieve spontaneous circulation after cardiopulmonary resuscitation (CPR) survive until hospital discharge, with 14% experiencing neurological disability [7]. These statistics underscore the critical importance of prompt diagnosis and appropriate management of IHCA to enhance survival rates and mitigate neurological sequelae [4]. Given that IHCA patients often present with multiple comorbidities, their overall survival rates are lower. Furthermore, individuals with more severe clinical conditions are at heightened risk of IHCA and subsequent multi-organ failure and neurological complications [8]. Consequently, they require extensive care either at home or in long-term care facilities, imposing a substantial burden on both families and society [9].

Studies have revealed the challenges faced by medical personnel in accurately predicting favorable neurological outcomes following cardiac arrest (CA) [10, 11]. Consequently, there is a pressing need for medical professionals to forecast outcomes objectively before CA occurs. However, existing predictive tools have demonstrated limited efficacy in assessing survival rates post IHCA. As a result, treatment decisions, particularly those made by physicians, often rely heavily on clinical judgment [12, 13]. This underscores the necessity for a robust predictive tool capable of accurately forecasting patient prognosis following cardiopulmonary arrest. Such a tool would serve as a critical foundation for formulating treatment and care objectives [14].

In 2012, Chan et al. introduced the CASPRI (Cardiac Arrest Survival Post Resuscitation In-Hospital) score for predicting survival with favorable neurological outcomes based on data from 42,957 patients across 551 hospitals. This tool incorporates key elements of the chain of survival [15]. Validated initially in an Asian population [16], CASPRI has demonstrated high effectiveness in predicting both survival rates and discharge with favorable neurological outcomes following in-hospital cardiac arrest [17]. The GO-FAR (Good Outcome Following Attempted Resuscitation) score was developed based on data from 51,240 patients across 366 hospitals between 2007 and 2009 who experienced in-hospital cardiac arrest [14]. Subsequently, in 2019, the PIHCA (The Prediction of outcome for in-Hospital Cardiac Arrest) score was introduced, utilizing variables from GO-FAR and data from 717 patients with in-hospital cardiac arrest in Sweden. PIHCA aimed to minimize discrepancies with GO-FAR by refining certain variables and enhancing their interpretational clarity [18].

The presence of a reliable predictive score within hospital settings can serve as a valuable resource for medical staff, aiding in the accurate anticipation of resuscitation outcomes and subsequent consequences [11]. In Iran, the absence of such predictive tools has created a significant knowledge gap regarding the impact of predictive factors on rehabilitation outcomes and their objective aftermath. Moreover, in adherence to Islamic culture, non-supportive treatment and the implementation of DNR (Do Not Resuscitate) orders are prohibited. The introduction of effective predictive scores tailored to the Iranian context holds promise for initiating a comprehensive program aligned with ethical principles, facilitating the selection of optimal treatment and care pathways, and alleviating the physical and psychological burden on medical staff. Therefore, this study aims to address the aforementioned knowledge gap by validating three scores—CASPRI, GO-FAR, and PIHCA—within the Iranian context.

## Methods

### Study design and setting

This cohort study is a retrospective analysis conducted using data extracted from the archives and electronic records of resuscitated patients admitted to three educational and therapeutic hospitals affiliated with Qazvin University of Medical Sciences, Qazvin, Iran. The data covers the period from 00:00 on March 21, 2018, to 24:00 on March 20, 2023.

These three hospitals collectively serve a large patient population, with approximately 54,000 patients annually and a combined total of 650 active beds. They serve as the primary centers for trauma, cardiac, neurological, and internal medicine care in Qazvin province, attracting a significant portion of the region's leading medical faculty. The hospitals' resuscitation teams comprise a designated resuscitation physician, an anesthesiologist, and ward nurses trained in resuscitation protocols. These teams respond promptly to Code 99 alerts, mobilizing to the patient's bedside upon notification by the attending nurse. Notably, extracorporeal cardiopulmonary resuscitation (ECPR) was not utilized in any of the cases studied.

### Study population

The inclusion criteria were age over 18 years and in-hospital cardiac arrest. Exclusions were made for prison inmates, individuals with mental disorders, pregnant women, patients with HIV, cases of out-of-hospital cardiac arrest, and patients transferred to another facility following resuscitation. Data pertaining to each CA case was gathered from electronic records and archived documents using a structured form designed in accordance with the Utstein style. For each patient, assessments using the CASPRI, GO-FAR, and PIHCA scores were conducted. The accuracy of the collected information was validated by two members of the study team, both faculty members of the Special Care Department at Qazvin University of Medical Sciences. Subsequently, the predictive power of neurological outcomes was compared across the three scores.

### Independent variables

In addition to the aforementioned scores, this study also analyzed data on variables including age, gender. heart problems, conditions before cardiac arrest (neurologically intact at admission), pre arrest CPC score, non- cardiac admission, CCI, respiratory insufficiency, mechanical ventilation, renal insufficiency, hypotension or hypoperfusion, nosocomial infection, use of vasopressors, initial rhythm of cardiac arrest (VT/VF, PEA, Asystole), location (CCU, ICU/EMS/General), duration of resuscitation, cardiac arrest shift.

### Outcomes

The main outcome of this study is the favorable neurological outcome after in-hospital cardiac arrest.

## The CASPRI score

Factors such as CPR duration, neurological status 24 h pre-cardiac arrest, location of cardiac arrest, mechanical ventilation, kidney dysfunction, liver dysfunction, sepsis, malignancy, and patient hypotension are utilized to predict survival with favorable neurological outcomes. The tool generates a final score ranging from 0 to 100. The likelihood of survival with a favorable neurological outcome correlates with the tool's score as follows: score 0–4 (82.6%), score 5–9 (66.6%), score 10–14 (42%), score 15–19 (23.1%), score 20–24 (12.3%), score 25–29 (5.2%), score 30–34 (2.1%), and score 35–40 (0%). A score of 40 indicates a 0% likelihood of survival with favorable neurological outcomes following cardiopulmonary resuscitation [15].

### The GO-FAR score

The GO-FAR tool has 13 variables. Central to this tool is the evaluation of consciousness state GCS 15 (Glasgow coma scale) upon admission and CPC 1(cerebral performance category) At the time of discharge, identified as the primary predictor of optimal neurological function. Poor prognosis is associated with factors such as severe trauma, stroke, malignancy, sepsis, and hospitalization for non-cardiac medical conditions. Additionally, predictors include liver dysfunction, admission from skilled nursing facilities, hypotension, renal dysfunction or dialysis, respiratory failure, pneumonia, and age [14]. The final tool score falls within the range of 11–15, with survival probability categorized into four groups: score 6 to -15 (above 15%), score -5 to 13 (3–15%), score 14–23 (less than 3%), and score equal to or greater than 24 (less than 1%).

### The PIHCA score

Variables in the PIHCA tool encompass age, neurological function status at discharge (CPC1-2), sepsis, pneumonia, hypotension, respiratory failure, hospitalization for non-cardiac diseases, acute renal failure, and the Charlson Comorbidity Index (CCI). Notable differences from the GO-FAR tool include the inclusion of CCI as an independent predictor, removal of trauma and stroke due to their low prevalence, exclusion of cancer and liver failure due to their incorporation into CCI, and redefinition of variables such as blood pressure, respiratory failure, renal failure, and CPC based on updated guidelines. The item regarding admission from skilled nursing facilities was omitted due to societal differences in Sweden's healthcare system structure [18]. Scoring methodology aligns with the GO-FAR tool, but the inclusion of CCI results in variable final scores. Unlike the GO-FAR tool, the probability of favorable neurological survival is categorized into three groups: very low probability (< 1%), low probability (1–3%), and above low probability (> 3%) [18].

### CPC score

CPC (Cerebral Performance Category) is a five-level scale to assess neurological function after cardiac arrest. It is a qualitative scale initially developed by Jennett et al. in 1975. The CPC score is a five-point scale that ranges from good brain function (level 1) to brain death (level 5) and is usually divided into two groups: "favorable" (CPC 1–2) versus "unfavorable" (CPC 3–5).

- CPC 1: Good cerebral performance or partial neurological impairment.
- CPC 2: Moderate cerebral disability.
- CPC 3: Severe cerebral disability, requiring complete assistance for all activities.
- CPC 4: Coma or vegetative state.
- CPC 5: Brain death.

This scale has been used as a primary or secondary outcome in research studies and quality improvement [19].

### Ethical considerations

This study was conducted by the Declaration of Helsinki and was approved by the ethics committee of Qazvin University of Medical Sciences with the code (IR.QUMS.REC. 1401.349). Due to the retrospective and observational nature of this low-risk study, the requirement to obtain informed consent from all subjects by the researcher was canceled by the national regulations for conducting retrospective research. Analysis was performed using anonymized clinical data to ensure privacy and confidentiality. All identifiable patient information was removed before analysis. For reporting, the principles of the Strengthening the Reporting of Observational Studies in Epidemiology (STROBE) guidelines were followed.

### Statistical analyses

Data analysis was performed using SPSS version 25 (Armonk, NY: IBM Corp) and Stata 11 (StataCorp LP. College Station). Descriptive statistics including mean and standard deviation, median and interquartile range, and frequency and percentage were used to describe the data. Qualitative variables were compared between two groups with favorable and unfavorable performance using the Chi-square test or Fisher's exact test, and continuous variables were compared with Mann–Whitney test. The multivariable analysis of the logistic regression model was used to investigate the predictors of the desired neurological outcome performance. Variables with *P*-value > 0.2 in the univariable analysis were included in the final multivariable logistic regression model. Odds Ratio (OR) and 95% Confidence Interval (95% CI) was estimated. Determining the accuracy and prediction of adverse neurological function based on the score of CARPRI, GO-FAR, and PIHCA tools in patients with cardiopulmonary resuscitation was performed by analyzing the roc curve (area under the receiver operating characteristics curve (AUROCC). Youden's index (sensitivity + specificity—1) was used to determine the cut point of the tools. Sensitivity, specificity, Positive predictive value (PPV), Negative predictive value (NPV) was estimated based on the determined cut point. We used DeLong test in Med Calc to evaluate differences between tools. The significance level of all tests is considered less than 0.05.

## Results

### Clinical characteristics and outcomes

**Fig. 1:**
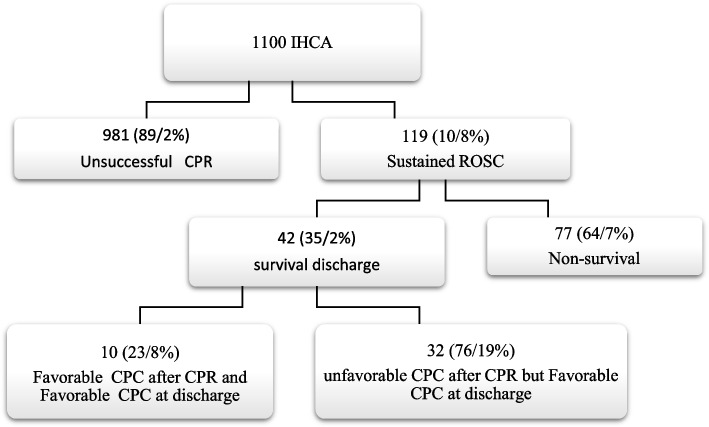
IHCA In-hospital cardiac arrest, CPR cardiopulmonary resuscitation, ROSC return of spontaneous circulation, CPC cerebral performance category

Among the 1100 in-hospital cardiac arrests studied, 981 individuals (89.2%) experienced unsuccessful cardiopulmonary resuscitation, while 119 (10.8%) achieved spontaneous circulation within 24 h. Only 42 individuals (35.2%) with spontaneous circulation and CPC [1, 2] survived to discharge, resulting in a study-wide survival rate of 3.8% (Fig. 1). The median age of all participants was 65, with survivors median 55 years and deceased individuals median 66 years. The majority of participants were male (64.5%) (Table 2). In the survival group, 37 individuals (88.1%) had a CPC score of [1, 2] 24 h prior to cardiac arrest, while in the deceased group, 997 individuals (94.2%) had a CPC score of ≥ 3 during the same period. The study findings revealed significant associations between neurological condition upon hospital admission, pre-cardiac arrest hospital infections, onset rhythm of cardiac arrest, duration of resuscitation, resuscitation shift, and attending physician, and post-resuscitation prognosis (*P* < 0.05) (Table 2). Detailed patient characteristics and resuscitation conditions are summarized in Tables 1 and 2.

**Table 1:** Characteristics of patients with and without return of spontaneous circulation

	**survival (42)**	**non-survival (1058)**	**Total (1100)**	*P* value*
Age (median, IQR)	55.00 (45.00–65.00)	66.00 (48.00–77.00)	65.00 (48.00–77.00)	0.002
Gender (Male)	30 (71.4%)	680 (64.3%)	710 (64.5%)	0.342
heart problems	33 (78.6%)	602 (56.9%)	635 (57.7%)	0.005
Conditions before cardiac arrest				
Neurologically intact at Admission	27 (64.3%)	19 (1.8%)	46 (4.2%)	< 0.001
Prearrest CPC score				< 0.001
CPC 1	1 (2.4%)	1 (0.1%)	2 (0.2%)	
CPC 2	36 (85.7%)	60 (5.7%)	96 (8.7%)	
CPC ≥ 3	5 (11.9%)	997 (94.2%)	1002 (91.1%)	
Non- Cardiac admission	8 (19%)	1003 (94.8%)	1011 (91.9%)	< 0.001
CCI (median, IQR)	2 (2–2.25)	2 (0–4)	2 (0–4)	0.108
Respiratory insufficiency	6 (14.3%)	1014 (95.8%)	1020 (92.7%)	< 0.001
Mechanical ventilation	4 (9.5%)	723 (68.3%)	727 (66.1%)	< 0.001
Renal insufficiency or dialysis	2 (4.8%)	332 (31.4%)	334 (30.4%)	< 0.001
Hypotension or hypoperfusion	7 (16.7%)	566 (53.5%)	573 (52.1%)	< 0.001
Nosocomial infection				0.001
UTI	1 (2.4%)	72 (6.8%)	73 (6.6%)	
SEPSIS	0 (0%)	13 (1.2%)	13 (1.2%)	
VAP	0 (0%)	91 (8.6%)	91 (8.3%)	
Infection due to pressure sores	0 (0%)	2 (0.2%)	2 (0.2%)	
More than one Nosocomial infection	0 (0%)	92 (8.7%)	92 (8.4%)	
Use of vasopressors	7 (16.7%)	556 (52.6%)	563 (51.2%)	< 0.001
*CRF* Chronic renal failure, *CPC* Cerebral Performance Category, *CCI* Charlson Comorbidity Index, *UTI* Urinary Tract Infection, *VAP* Ventilator-associated pneumonia				

^*^Chi Square or Mann-Whitney test

**Table 2:** Factors associated with CPR and neurological outcome

	**Survival (42)**	**Non-survival (1058)**	**Total (1100)**	*P* value^*^
Initial rhythm of cardiac arrest				
VT/VF	37 (88.1%)	34 (3.2%)	71 (6.5%)	
PEA	0 (0%)	416 (39.3%)	416 (37.8%)	
Asystole	5 (11.9%)	608 (57.5%)	613 (55.7%)	
Location				< 0.001
CCU	31 (73.8%)	848 (80.2%)	879 (79.9%)	
ICU/EMS/General	11 (26.2%)	210 (19.8%)	221 (20.1%)	
Duration of resuscitation, min				< 0.001
0–4	31 (73.8%)	9 (0.9%)	40 (3.6%)	
5–9	8 (19%)	5 (0.5%)	13 (1.2%)	
10–14	1 (2.4%)	0 (0%)	1 (0.1%)	
15–29	2 (4.8%)	0 (0%)	2 (0.2%)	
≥ 30	0 (0%)	1044 (98.6%)	1044 (94.9%)	
Cardiac arrest shift				0.007
Morning	15 (35.7%)	305 (28.8%)	320 (29.1%)	
Evening	19 (45.2%)	302 (28.5%)	321 (29.2%)	
Night	8 (19%)	451 (42.6%)	459 (41.7%)	
CASPRI (median, IQR)	5 (3–8.25)	31 (28–35)	31 (28–35)	< 0.001
GO-FAR (median, IQR)	-13 (-15–2.75)	24 (20–30)	23 (19–29)	< 0.001
PIHCA (median, IQR)	-12 (-13- -7)	19 (12–28)	19 (11–27)	< 0.001

*VF* ventricular fibrillation, *VT* ventricular tachycardia, *PEA* pulseless electrical activity, *CPR* cardiopulmonary resuscitation, *CASPRI* cardiac arrest survival post resuscitation in-hospital, *GO-FAR* good outcome-following attempted resuscitation, PIHCA the Prediction of outcome for In-Hospital Cardiac Arrest

*Chi Square or Mann-Whitney test

### The accuracy of CASPRI, GO-FAR, and PIHCA scores in predicting adverse neurological outcomes

The overall median CASPRI score was 31, with a median of 31 in the deceased group and 5 in the surviving group. The mean GO-FAR score was 23 overall, with a mean of 24 in the deceased group and -13 in the survival group. Similarly, the overall median PIHCA score was 19, with a median of 19 in the deceased group and -12 in the survival group. The predictive performance of CASPRI, GO-FAR, and PIHCA scores for adverse neurological function, as measured by the area under the ROC curve, is illustrated in Fig. 1.

The chance of adverse neurological outcomes increased by 40% with each unit increase in CASPRI score (OR = 1.40, 95% CI, 1.31 to 1.51), by 21% with each unit increase in GO-FAR score (OR = 1.21, 95% CI, 1.17 to 1.21), and 28% with each unit increase in PIHCA score (OR = 1.28, 95% CI, 1.21 to 1.35).

AUC for the CASPRI score was 0.99 (95% CI, 0.98–1.00), for the GO-FAR score was 0.98 (95% CI, 0.97–0.99), and for the PIHCA score was 0.96 (95% CI, 0.94–0.99).

Based on the Youden index, the cutoff point values were determined to be 22.5 for CASPRI (sensitivity = 94.6% (95% CI, 93.1%, 95.9%), specificity = 97.6% (95% CI, 87.4%, 99.9%), PPV = 99.9% (95% CI; 99.4%, 100%), NPV = 41.8% (95% CI, 31.9%, 52.2%)).

Cutoff were determined to be 5.5 for GO-FAR (sensitivity = 97.1% (95% CI, 95.9%, 98%), specificity = 85.7% (95% CI, 71.5%, 94.6%) PPV = 99.4% (95% CI; 98.7%, 99.8%), NPV = 53.7% (95% CI, 41.1%, 66.0%)). Also, cutoff were determined to be 1.5 for PIHCA (sensitivity = 93.6% (95% CI, 91.9%, 95%), specificity = 90.5% (95% CI, 77.4%, 97.3%), PPV = 99.6% (95% CI; 99.0%, 99.9%), NPV = 35.8% (95% CI, 26.8%, 45.7%)).

**Fig. 2:**
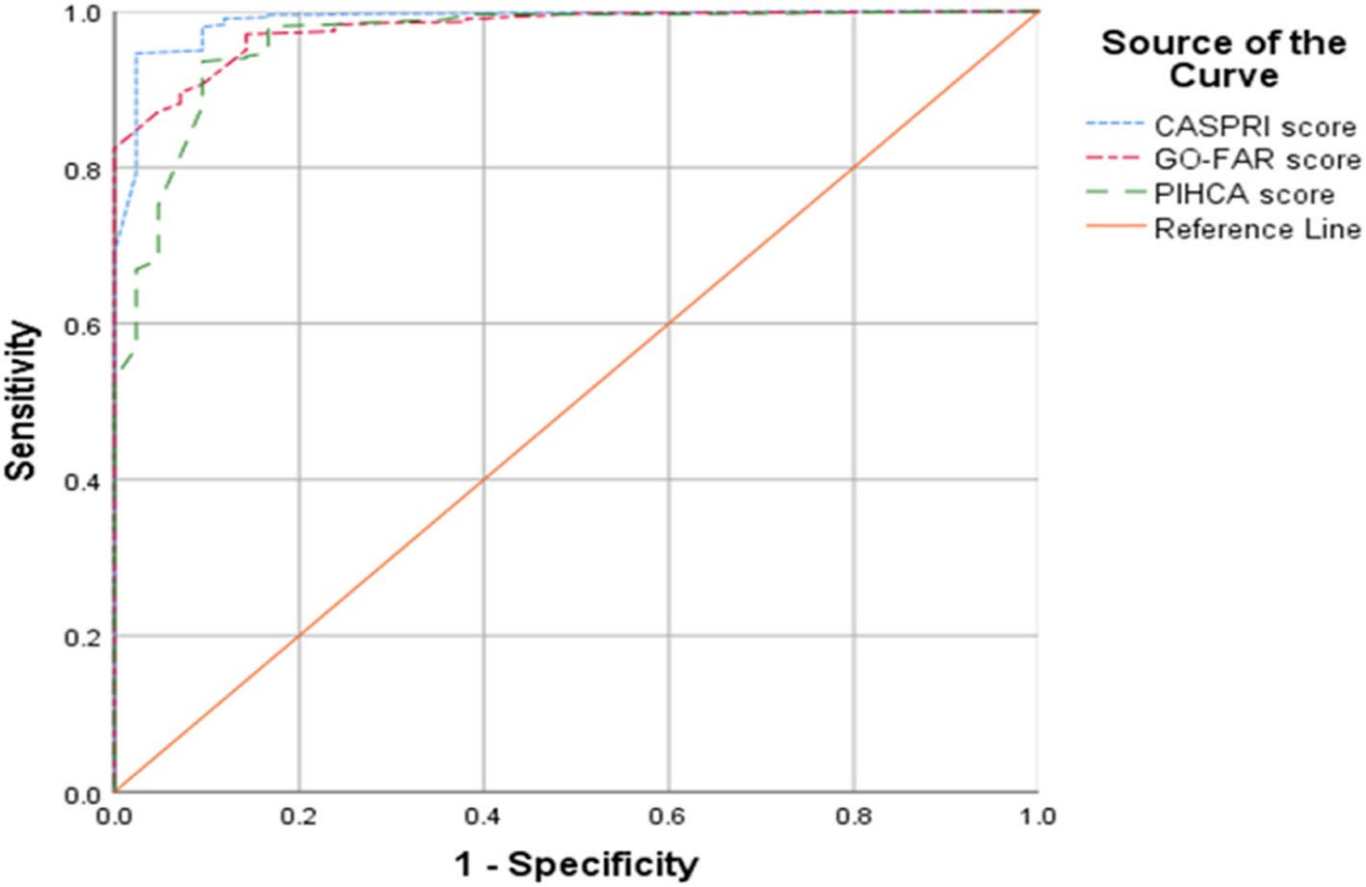
ROC curve for predicting adverse neurological outcomes based on CASPRI, GO-FAR and PIHCA tools. Area under curve (AUC) for CASPRI score; 0.99 (95% CI, 0.98–1.00), AUC for GO-FAR score; 0.98 (95% CI, 0.97–0.99), AUC for PIHCA score; 0.96 (95% CI, 0.94–0.99). CASPRI: cardiac arrest survival post resuscitation in-hospital, GO-FAR; good outcome-following attempted resuscitation. PIHCA: the Prediction of outcome for In-Hospital Cardiac Arrest

Results of the DeLong et al. test revealed a significant difference in predictive power between CASPRI and PIHCA tools (*P* = 0.001), while no significant difference was observed between CASPRI and GO-FAR (*P* = 0.057). Additionally, there was no significant difference between the predictive abilities of GO-FAR and PIHCA tools (*P* = 0.159). (Fig. 2).

### Factors associated with favorable neurological outcomes in in-hospital cardiac arrest

Multivariable logistic regression analysis was conducted to examine factors associated with favorable neurological outcomes in IHCA. The analysis revealed that advanced age, respiratory failure, night shift compared to morning shift, renal failure, and prolonged resuscitation were associated with poor neurological prognosis (Table 3). Additionally, a CPC score recorded 24 h before cardiac arrest (≥ 3 vs 1 and 2) was found to be predictive of neurological prognosis post-resuscitation. In univariable analysis, neurological function status upon admission exhibited a high odds ratio (OR 98.432, 95% CI 45.242–214.155). However, in multivariate analysis, after accounting for various factors, the odds ratio reduced to (OR 0.431, 95% CI 0.055–3.406) (Table 3).

**Table 3:** Univariable and Multivariable logistic regression for predicting favorable neurologic outcome

	**univariable**		**Multivariable**	
Variables	**Odd ratio (95% Cl)**	**p**	**Odd ratio (95% Cl)**	**p**
Age	0.982 (0.968–0.996)	0.012	0.956 (0.914–0.999)	0.043
CPC before Arrest 1, 2	Reference			
CPC before Arrest ≥ 3	0.008 (0.003–0.022)	< 0.001	0.055 (0.006–0.476)	0.008
Respiratory insufficiency	0.007 (0.003–0.018)	< 0.001	0.043 (0.004–0.456)	0.009
Vasopressor use	0.181 (0.079–0.410)	< 0.001	4.177 (0.146- 119.294)	0.403
Renal failure	0.105 (0.025–0.436)	< 0.001	0.104 (0.012–0.926)	0.042
Mechanical ventilation	0.049 (0.017–0.138)	< 0.001	1.659 (0.268- 10.274)	0.587
Location CCU	Reference			
ICU/EMS/General	0.016 (0.007–0.034)	< 0.001	0.403 (0.098- 1.661)	0.209
Neurological admission	98.432 (45.242–214.155)	< 0.001	0.431 (0.055- 3.406)	0.425
Nosocomial infection	0.071 (0.010–0.520)	0.009	0.494 (0.037- 6.541)	0.593
Heart problems	2.777 (1.316–5.862)	0.007	0.773 (0.076- 7.813)	0.827
Hypotension	0.173 (0.076–0.392)	< 0.001	0.064 (0.002- 1.980)	0.117
CPR duration	0.782 (0.746–0.818)	< 0.001	0.772 (0.691- 0.861)	< 0.001
Non- Cardiac admission	0.011 (0.005–0.025)	< 0.001	0.431 (0.055–3.406)	0.425
Morning shift	Reference		Reference	
Night shift	0.361 (0.151–0.861)	0.022	0.215 (0.050–0.931)	0.040
CCI	0.872 (0.743–1.022)	0.091	0.564 (0.303- 1.050)	0.071

*CPR* cardiopulmonary resuscitation, *CPC* Cerebral Performance Category, *CCI* Charlson Comorbidity Index

## Discussion

Predicting the favorable neurological outcome after IHCA is a major concern for the treatment staff and patients' families. Because it can guide the path of subsequent treatment decisions and have a significant impact on the quality of life of the patient and their family.

Comparison of the AUC using the DeLong et al. test revealed a *P* value of 0.057 between the CASPRI and GO-FAR tools, 0.001 between the PIHCA and CASPRI tools, and 0.159 between the PIHCA and GO-FAR tools. These results indicate a significant difference in predictive power between the PIHCA and CASPRI tools. However, while there is a significant difference in the predictive ability of CASPRI compared to PIHCA and GO-FAR, the overlap of their confidence intervals suggests that CASPRI performs significantly better in predicting favorable neurological outcomes.

Therefore, it can be inferred that CASPRI is a relatively suitable model for predicting neurological outcomes post-resuscitation compared to the other two tools. One of the distinguishing features of CASPRI is its consideration of conditions both before and during resuscitation, whereas the other two tools solely focus on pre-resuscitation conditions. This distinction underscores the importance of evaluating the characteristics of each tool in predictive modeling.

In a study conducted by Wang et al. [16] in Taiwan, the area under the curve (AUC) of the CASPRI tool was reported as 0.79. Similarly, Jung et al. [19] reported an AUC of 0.75 and a survival rate of 20.8%. Additionally, Chou et al. [20] conducted a study in the emergency department of Taiwan, reporting an AUC of 0.81 and a survival rate of 20.5%. Similarly, Tsai et al. [21] reported an AUC of 0.77. However, in the present study, an AUC of 0.99 was reported, which is notably higher than the AUC reported in previous studies.

Also, in the study of Jung et al. [19], the AUC of the GO-FAR tool was 0.67, in the study of Thai et al. [22] 0.75, in the study of Piscator et al. [18] 0.82, in the study of Ohlsson et al. [8] 0.85, and in the study of Cho and Colleagues [23] reported a survival rate of 0.81% and a survival rate of 25.4%, which compared to our study, the survival rate was higher and the AUC was lower.

Finally, in Piscator, et al.'s study [18], which is the only study that compares the PIHCA tool with the GO-FAR tool, the AUC of the PIHCA tool was reported as 0.80, while this number in our study was 0.96, which is higher than this study.

While both CASPRI and GO-FAR scores offer utility, their applications differ. CASPRI is designed specifically for patients who have undergone successful cardiopulmonary resuscitation, serving as an adjunctive tool to predict the likelihood of favorable neurological outcomes. On the other hand, the parameters of the GO-FAR score enable its use in all patients with critical clinical conditions preceding in-hospital cardiac arrest. It's important to emphasize that clinical decision-making should not solely rely on these two tools. Instead, they should be utilized as complementary aids alongside other paraclinical and clinical factors [19].

One plausible explanation for the variance in survival rates and predictive efficacy of the three tools observed in this study compared to previous research may stem from the illegality of the DNR order in Iranian society, stemming from its conflict with Iranian Islamic cultural norms. This legal constraint may contribute to an increase in the number of unsuccessful resuscitations, as all patients are subjected to resuscitation efforts regardless of the severity of their condition. Previous studies, guided by the ethical principle of autonomy, have highlighted the patient's right to participate in healthcare decisions, including those related to DNR orders [18, 24, 25]. Additionally, research indicates that knowledge of a patient's preferences regarding CPR can significantly impact prognostic outcomes, posing a challenge for medical staff in predicting post-cardiac arrest outcomes [10, 26, 27]. Furthermore, the variability in disease categories and clinical department settings, coupled with varying access levels to multidisciplinary treatments and specialized care teams across hospitals, may hinder the implementation of uniform and integrated post-cardiac arrest treatment protocols [19].

Furthermore, in previous studies, questions regarding the necessity of treatments following cardiac arrest for patients with incurable diseases or irreversible clinical deterioration have been raised due to limited life expectancy. However, in Iran, treatment orders are executed in accordance with principles of benevolence and justice, and the absence of DNR orders for all patients, irrespective of disease severity, further influences treatment decisions.

Moreover, variations in study environments and populations may also contribute to observed differences. Unlike many similar studies conducted in single centers and focusing on specific patient groups within certain departments, our study took a more comprehensive approach, encompassing three centers with diverse patient admissions spanning various disease types. Additionally, similar to the study by Cho et al., our study considered a CPC score of 1 or 2 as indicative of favorable neurological outcomes upon discharge.

Ultimately, the discrepancies in AUC values among the tools can be attributed to differences in sampling conditions and study objectives across various research endeavor.

Moreover, multivariate regression analysis conducted in this study highlighted the significant impact of several factors on favorable neurological outcomes. Notably, maintaining a favorable neurological function status 24 h before resuscitation and a younger age emerged as crucial contributors to positive outcomes. Conversely, the presence of respiratory failure, kidney failure, occurrence of cardiac arrest during the night shift, and prolonged resuscitation were associated with poorer neurological outcomes.

Collectively, these findings offer valuable insights into assessing neurological prognosis and informing decision-making regarding in-hospital resuscitation protocols.

The neurological performance status 24 h preceding resuscitation significantly influenced neurological outcomes upon discharge, a finding consistent with studies by Wang et al. [16] and Chan et al. [28]. It appears that lower CPC scores prior to cardiac arrest correlate with a higher likelihood of favorable neurological outcomes post-resuscitation. Furthermore, patients with pre-existing ischemic or hemorrhagic brain changes before cardiac arrest tend to experience more adverse neurological outcomes afterward, with the extent of pre-existing damage serving as a significant predictor of post-resuscitation neurological outcomes [29]. Some studies have reported that only a minority of individuals with impaired brain function survived cardiac arrest, often experiencing severe disabilities [30–33]. Hence, meticulous monitoring of even minor neurological changes, preventive measures against severe injuries, and timely interventions can profoundly influence cardiac arrest outcomes.

Age emerges as another predictor of favorable neurological outcomes in this study. Existing research suggests that younger individuals are more likely to survive with favorable neurological outcomes following cardiopulmonary arrest [11, 15, 16, 19]. However, conflicting results have been reported in several studies, indicating that age may not necessarily impact neurological outcomes and survival rates [18, 21].

Advancing age is associated with increased susceptibility to conditions such as heart failure, stroke, and kidney dysfunction. Consequently, the complications arising from these conditions pose a significant threat to favorable neurological outcomes post-resuscitation, particularly in elderly individuals [34].

The duration of resuscitation also correlates with neurological outcomes following resuscitation efforts. Several studies have consistently shown that a shorter duration of time to achieve spontaneous circulation is associated with a higher likelihood of survival with favorable neurological outcomes [16, 19, 21].

The presence of renal failure before cardiac arrest emerges as a predictor associated with poor neurological outcomes following successful cardiopulmonary resuscitation. This finding aligns with results from studies by Tamura et al. [35], Wang et al. [16], and Tsai et al. [21], which also reported similar outcomes. However, Cassina et al. [36] found that kidney dysfunction did not significantly impact results in their study.

Several potential explanations may elucidate this association. Following restoration of spontaneous circulation (ROSC), the brain becomes vulnerable to hemodynamic disturbances due to the loss of its natural self-regulation ability and microvascular perfusion disruption [37]. Consequently, cerebral perfusion may be more compromised in post-cardiac arrest patients with renal dysfunction, attributed to underlying cardiovascular dysfunction, thereby establishing a link between renal function and neurological outcomes post-cardiac arrest. Additionally, some patients may experience acute kidney injury (AKI) before and/or after cardiac arrest, further complicating the scenario [35].

AKI may contribute to premature death through mechanisms such as acute elevation of inflammatory cytokines and endothelial dysfunction [38]. Interventions aimed at preserving kidney function may potentially influence the outcome of cardiac arrest. Therefore, there is clinical significance in prioritizing the overall health of patients with renal dysfunction who do not require hemodialysis.

The presence of respiratory failure before cardiac arrest is identified as another predictor associated with favorable neurological outcomes. One study has demonstrated that lung function may mitigate nerve damage, and recovery from acute respiratory distress syndrome (ARDS) can reduce brain damage [39]. However, conflicting findings exist, with some studies reporting no significant relationship between these two factors [19, 21].

ARDS, a common cause of respiratory failure, significantly increases mortality rates, particularly in critically ill patients [40]. Mechanical ventilation is crucial for managing ARDS, yet it can potentially induce lung parenchymal damage through excessive alveolar overdistension [41–43]. Pulmonary compliance serves as a key indicator of ARDS severity, with studies indicating that higher pulmonary compliance is associated with improved neurological outcomes post-resuscitation [44].

Severe ARDS levels can exacerbate oxygen and carbon dioxide level imbalances, leading to brain damage [44, 45]. Patients with reduced lung compliance are at higher risk of unfavorable neurological outcomes due to ventilator-induced lung injuries such as barotrauma [46]. Therefore, it is advisable for healthcare providers to adhere to guidelines aimed at enhancing pulmonary compliance to mitigate complications associated with respiratory failure and improve neurological outcomes following resuscitation.

The findings of this study indicate that experiencing cardiac arrest during the night shift, in contrast to the morning shift, is linked to adverse neurological consequences. Consistent with these results, several studies have highlighted that patients who suffer cardiac arrest during nighttime or weekends tend to experience severe complications, although some are successfully revived after resuscitation [34, 47–49]. Conversely, other studies have reported no significant impact of cardiac arrest occurrence during specific shifts on resuscitation outcomes [3].

Several factors may contribute to this situation, including the slowed emergency response rates due to fatigue, reduced staffing levels during nighttime shifts, and potentially higher occurrences of unwitnessed arrests during nighttime hours [34]. Additionally, the misalignment between nurses' chronotype and the demands of their work, potentially impacting their performance, alertness, and productivity, could be another contributing factor [50–52]. Moreover, it is worth noting that in Iran, night shifts typically span 12 h, whereas in other countries, they often last for 8 h. The longer duration of night shifts in Iran may exacerbate nurse fatigue levels, potentially influencing their performance and productivity.

### Limitations

This study encountered several limitations. Firstly, the retrospective analysis of patient medical records may not fully capture their actual condition at the time of cardiac arrest, introducing potential discrepancies. Additionally, the incidence of in-hospital cardiac arrest reflects the disease burden among hospitalized patients, the availability of facilities to detect disease deterioration, and the efficacy of in-hospital resuscitation systems, all of which can vary across different hospital settings. Hence, multicenter studies with larger sample sizes are warranted to validate and corroborate the findings of this study.

Furthermore, while the Area Under the Receiver Operating Characteristic (AUROC) curve serves as a valuable tool for evaluating predictive ability, it may not fully account for demographic variations. Therefore, AUROC analysis should be complemented with other criteria and clinical insights to ensure a comprehensive assessment.

## Conclusion

In our study, the CASPRI and GO-FAR scores demonstrated promising potential for accurate prediction of IHCA outcomes within the Iranian population. The CASPRI score proves particularly useful for initial patient assessment immediately following successful cardiopulmonary resuscitation. Notably, five out of the eleven variables within the CASPRI score emerged as independent predictors of favorable neurological outcomes, findings that align with those observed in Eastern populations where DNR orders are commonplace. For patients with critical conditions, the GO-FAR score can be utilized as part of pre-cardiac arrest evaluation. Despite existing debates surrounding optimal therapeutic interventions and post-resuscitation care decisions, the insights garnered from our study hold significant value for guiding medical staff in navigating post-cardiac arrest syndrome management within Iranian healthcare settings.

### Clinical iplications and future research

#### Enhanced prediction of outcomes

Thus, CASPRI and GO-FAR are the fair prognostic score tools for the outcomes of IHCA in the Iranian population. Its application can improve the accuracy of outcomes predictions, and help clinicians to make the right decisions.

#### Improved initial assessment

It may be especially useful in the primary assessment of the patient on arrival to the emergency department after a successful CPR. This can lead to more precise and timely actions, better handling of the patients and possibly an increase in the patients’ prognosis.

#### Utility in pre-cardiac arrest evaluation

Looking at the advantages of the proposed GO-FAR score it can be seen that it is useful for assessing the health of patients with severe diseases that potentially. The GO-FAR score can be useful for the assessment of patients with critical conditions who might be at risk for cardiac arrest. It can be used in the pre-cardiac arrest assessment to optimize the allocation and disposition before such an occurrence.

#### Guidance for post-resuscitation care

As concluded in this study, more refined sort of care pattern after the occurrence of cardiac arrest applies in the Iranian context. The findings can help the medical staff to better understand the situation and improve the existing processes of patient’s management.

Future studies should cross-verify CASPRI and GO-FAR scores in varying cultures and environments to confirm the universality of these scale. Their effectiveness in forecasting outcomes and recovery needs to be evaluated in longitudinal researches. It should be possible to include other predictors to strengthen these scores and make a more precise diagnosis. Researchers should also assess the influence of these scores on the subsequent management and the post-cardiac arrest interventions plans; as well as compare the efficacy of these scores with others that are available.

## Supplementary Information

Supplementary Material 1.

Supplementary Material 2.

## Data Availability

No datasets were generated or analysed during the current study.

## Abbreviations

IHCA: In-hospital cardiac arrest
OHCA: Out-of-hospital cardiac arrest
CA: Cardiac arrest
ECPR: Extracorporeal cardiopulmonary resuscitation
HIV: Human immunodeficiency virus
CASPRI: Cardiac Arrest Survival Post Resuscitation In-Hospital
CCI: Charlson Comorbidity Index
GO-FAR: Good outcome-following attempted resuscitation
PIHCA: The Prediction of outcome for In-Hospital Cardiac Arrest
DNR: Do Not Resuscitate
ROSC: Restoration of spontaneous circulation
AKI: Acute kidney injury
ARDS: Acute respiratory distress syndrome
AUROC: Area Under the Receiver Operating Characteristic
CRF: Chronic renal failure
CPC: Cerebral Performance Category
UTI: Urinary Tract Infection
VAP: Ventilator-associated pneumonia
VF: Ventricular fibrillation
VT: Ventricular tachycardia
PEA: Pulseless electrical activity
CPR: Cardiopulmonary resuscitation
