# Supplementary material for: Validation of CASPRI, GO-FAR, PIHCA scores in predicting favorable neurological outcomes after in-hospital cardiac arrest; A five-year three center retrospective study in IRAN
Source: BMC Cardiovasc Disord. 2024 Oct 29;24:603. doi: 10.1186/s12872-024-04229-8 (PMC11520468; doi:10.1186/s12872-024-04229-8)
Supplement: Supplementary file 2 — Supplementary Material 2. [file 12872_2024_4229_MOESM2_ESM.docx]

**CASPRI Score**

| Predictor Point | Predictor Point |
| --- | --- |
| 1) Age Group, yrs  <50 0  50-59 0  60-69 1  70-79 2  ≥80 4 | 4) Hospital location  Telemetry unit 0  Intensive care 1  Non-monitored unit 3 |
| 2) Initial Arrest Rhythm  VF/VT, Time to Defibrillation  ≤ 2 minutes 0  3 minutes 0  4-5 minutes 2  >5 minutes 3  Pulseless electrical activity 6 Asystole 7 | 5) Duration of resuscitation, min  <2 0  2-4 0  5-9 3  10-14 5  15-19 6  20-24 6  25-29 6  ≥30 8 |
| 3) Prearrest CPC score  1 0  2 2  3 9  ≥4 9 | Factors present prior to arrest  6) Mechanical ventilation 3  7) Renal insufficiency 2  8) Hepatic insufficiency 4  9) Sepsis 3  10) Malignant disease 4  11) Hypotension 3 |

**GOFAR Score**

| GO-FAR  Score | Variable |
| --- | --- |
| -15 | Neurologically intact or with minimal deficits at  Admission (CPC 1) |
| +10 | Major trauma |
| +8 | Acute stroke |
| +7 | Metastatic or hematologic cancer |
| +7 | Septicemia |
| +7 | Medical noncardiac diagnosis |
| +6 | Hepatic insufficiency |
| +6 | Admit from skilled nursing facility |
| +5 | Hypotension or hypoperfusion |
| +4 | Renal insufficiency or dialysis |
| +4 | Respiratory insufficiency |
| +1 | Pneumonia |
|  | Age, y |
| 0 | 70< |
| +2 | 70-74 |
| +5 | 75-79 |
| +6 | 80-84 |
| +11 | 85≤ |

**PIHCA Score**

| **PIHCA Score** | **Predictors** |
| --- | --- |
| -15 | Neurologically intact at  Admission (CPC 1-2) |
| +7 | Sepsis |
| +1 | Pneumonia |
| +5 | Hypotension |
| +4 | Respiratory insufficiency |
| +7 | Medical non-cardiac  Admission |
| +4 | Acute Kidney Injury |
|  | CCI |
|  | Age, y |
| 0 | 70> |
| +2 | 70-74 |
| +5 | 75-79 |
| +6 | 80-84 |
| +11 | 85≤ |

**CPC score(Cerebral Performance Category)**

| Cerebral Performance Category | Standard Definition |
| --- | --- |
| CPC 1 | Good cerebral performance: conscious, alert, able to work, might have mild neurologic or psychological deficit. |
| CPC 2 | Moderate cerebral disability: conscious, sufficient cerebral function for independent activities of daily life. Able to work in a sheltered environment. |
| CPC 3 | Severe cerebral disability: conscious, dependent on others for daily support because of impaired brain function. Ranges from ambulatory state to severe dementia or paralysis. |
| CPC 4 | Coma or vegetative state: any degree of coma without the presence of all brain death criteria. Unawareness, even if appears awake (vegetative state) without interaction with environment; may have spontaneous eye opening and sleep/awake cycles. Cerebral unresponsiveness. |
| CPC 5 | Brain death: apnea, areflexia, EEG silence, etc. |
